# An Antivirulence Approach for Preventing Cryptococcus neoformans from Crossing the Blood-Brain Barrier via Novel Natural Product Inhibitors of a Fungal Metalloprotease

**DOI:** 10.1128/mBio.01249-20

**Published:** 2020-07-21

**Authors:** Phylicia A. Aaron, Kiem Vu, Angie Gelli

**Affiliations:** a Department of Pharmacology, School of Medicine, University of California, Davis, California, USA; Duke University Medical Center

**Keywords:** high throughput screen, metalloprotease, Mpr1, blood-brain barrier, *Cryptococcus neoformans*, *Pichia pastoris*, natural products, phytochemical, cryptococcal meningitis

## Abstract

Cryptococcus neoformans (*Cn*) is the leading cause of fungal meningitis, a deadly disease with limited therapeutic options. Dissemination to the central nervous system hinges on the ability of *Cn* to breach the blood-brain barrier (BBB) and is considered an attribute of *Cn* virulence. Targeting virulence instead of growth for antifungal drug development has not been fully exploited despite the benefits of this approach. Mpr1 is a secreted fungal metalloprotease not required for fungal growth, but rather, it functions as a virulence factor by facilitating *Cn* migration across the BBB. This central role for Mpr1, its extracellular location, and lack of expression in mammalian cells make Mpr1 a high-value target for an antivirulence approach aimed at developing therapeutics for cryptococcal meningitis. To test this notion, we devised a large-scale screen to identify compounds that prohibited *Cn* from crossing the BBB by selectively blocking Mpr1 proteolytic activity, without inhibiting the growth of *Cn*. A phytochemical natural product-derived library was screened to identify new molecular scaffolds of prototypes unique to a *Cn* microecosystem. Of the 240 pure natural products examined, 3 lead compounds, abietic acid, diosgenin, and lupinine inhibited Mpr1 proteolytic activity with 50% inhibitory concentration (IC_50_) values of <10 μM, displayed little to no mammalian cell toxicity, and did not affect *Cn* growth. Notably, the lead compounds blocked *Cn* from crossing the BBB, without damaging the barrier integrity, suggesting the bioactive molecules had no off-target effects. We propose that these new drug scaffolds are promising candidates for the development of antivirulence therapy against cryptococcal meningitis.

## Abstract (precis)

Fungal infections like cryptococcal meningitis are difficult to resolve because of the limited therapies available. The small arsenal of antifungal drugs reflect the difficulty in finding available targets in fungi because like mammalian cells, fungi are eukaryotes. The limited efficacy, toxicity, and rising resistance of antifungals contribute to the high morbidity and mortality of fungal infections and further underscore the dire but unmet need for new antifungal drugs. The traditional approach in antifungal drug development has been to target fungal growth, but an attractive alternative is to target mechanisms of pathogenesis. An important attribute of Cryptococcus neoformans (*Cn*) pathogenesis is its ability to enter the central nervous system. Here, we describe a large-scale screen that identified three natural products that prevented *Cn* from crossing the blood-brain barrier by inhibiting the virulence factor Mpr1 without affecting the growth of *Cn*. We propose that compounds identified here could be further developed as antivirulence therapy that would be administered preemptively or serve as a prophylactic in patients at high risk for developing cryptococcal meningitis.

## INTRODUCTION

Mycotic diseases can be extremely serious and are often life-threatening for individuals with a compromised immune system. Every year 1.5 to 2 million deaths worldwide are attributed to fungal disease; however, these estimates are likely understated because of the lack of mandatory public health surveillance of fungal diseases (1). The challenges associated with treating fungal infections are largely due to the small repertoire of antifungals, their limited efficacy, their toxicity to the host, and the emergence of drug resistance. All of these factors combined have made it increasingly difficult to resolve fungal disease (2).

Among the most devastating diseases are fungal infections of the central nervous system (CNS) (3, 4). Although some antifungal agents can enter the CNS by penetrating the blood-brain barrier (BBB), it is nearly impossible to completely eradicate the fungus once it is discovered in the brain. Even following treatment, individuals will often experience severe neurological sequelae such as visual loss, cranial palsies, neurologic deficit, or mental impairment (5). The most common cause of fungal infections of the CNS continues to be Cryptococcus neoformans (*Cn*). Cryptococcal meningitis (CM) accounts for 15% to 20% of all HIV-related deaths (6, 7). In 2014, the global number of HIV-associated cases of CM was 223,100, resulting in 181,000 deaths (8). Deaths from non-HIV-related CM account for 1/4 of CM-related hospitalizations and 1/3 of CM-related deaths in the United States (6). In spite of access to antiretroviral therapy (ART), the incidence of CM remains high (9).

Treatment guidelines for HIV-associated CM recommend amphotericin B with flucytosine for 2 weeks as induction therapy, followed by an azole for a minimum of 10 weeks (10). Fifty percent of AIDS patients treated for CM will experience a relapse of the disease unless they receive maintenance therapy (10). Despite the superiority of this combination therapy, high acquisition costs of flucytosine restrict its availability in Africa and Asia, where the disease burden is the highest (11). In the absence of flucytosine, a combination of amphotericin B and fluconazole is the current guideline (10). Although they can be effective, these treatment plans have several drawbacks, including significant toxicity of amphotericin B and the intravenous administration requiring hospitalization and significant in-hospital care. Although fluconazole can penetrate the CNS, it is fungistatic and has poor fungal clearance even at high doses, and fluconazole resistance is on the rise (10, 12, 13).

The prevalence of *Cn* in the environment and its thermotolerance make it the perfect human pathogen. Once inhaled, spores of *Cn* proliferate in lung tissue and due to their highly neurotropic nature, disseminate to the CNS. Multiple steps make up the pathophysiology of CM, and many studies have identified several key factors that contribute to this process (14–27). A key protein required to successfully cross the BBB is Mpr1. Mpr1 belongs to a newly identified but poorly understood class of secreted metalloproteases (M36 fungalysins) found only in fungal pathogens (28–31). In the case of *Cn*, only one M36 fungalysin, *MPR1*, is present in its genome (31, 32). These secreted metalloproteases have a similar overall structure consisting of a signal peptide, a prodomain, and a catalytic domain that coordinates zinc and H_2_O for activity (33). So far, evidence for these metalloproteases support a role in fungal pathogenesis, but in most cases, their targets have not been identified (25). The crystal structure of the archetype of the M36 fungalysin (metalloprotease [MEP]) in Aspergillus fumigatus has been obtained (29). Structure studies revealed that MEP autoproteolyzes its N-terminal prodomain, releasing the C-terminal active polypeptide with its zinc-associated ligands (29).

Previous studies demonstrated that Mpr1 is not required for fungal viability/growth, but rather, it functions as a major virulence factor by promoting *Cn* across the BBB (23, 32, 33). We propose that Mpr1 is a high-value target based on studies that found significantly less brain fungal burden in mice that had been inoculated with a strain of *Cn* lacking Mpr1 (32). This discovery raised the notion that perhaps inhibition of Mpr1 activity could be a viable strategy for the development of antivirulence agents (34). Neutralizing virulence factors to disarm pathogens and rendering them less harmful or more easily susceptible to immune clearance has been gaining traction (34–40). Several studies have proposed the use of novel small-molecule inhibitors of fungal morphogenesis and biofilm formation as candidates for the development of antivirulence agents (41, 42). Others have demonstrated that the combination of antivirulence agents with existing antifungals can be synergistic (40). Therapies based on virulence factor inhibition could be envisioned as an adjunct to classical antifungals for severe/intractable infections or as prophylactic therapy for high-risk patients (40, 43). A clear advantage of antivirulence therapy is the lack of pressure for drug resistance to develop since fungal viability is not compromised. In addition, the antivirulence strategy would provide a much-needed pipeline for antifungal drug development.

In the case of Mpr1, its role as a virulence factor, extracellular location, and lack of expression in mammalian cells make it a highly desirable target. We tested this notion by developing an assay and used it to screen new compounds that could selectively block Mpr1 proteolytic activity without inhibiting the growth/viability of *Cn*. Of the pure natural products screened, three lead compounds inhibited the proteolytic activity of Mpr1 recombinant protein with 50% inhibitory concentration (IC_50_) values of <10 μM, they did not affect the growth/viability of *Cn*, and they showed little to no cytotoxicity to mammalian cells. Most notably, the three lead compounds prevented *Cn* from crossing the brain endothelial barrier without damaging the integrity of the barrier, suggesting that the compounds had no off-target effects. Our results validate the usefulness of a large-scale screen in identifying small-molecule inhibitors of Mpr1, and they support the proof-of-concept that Mpr1 is a high-value target for the development of antivirulence drugs.

## RESULTS

### Establishing parameters of the large-scale screen.

**FIG 1:**
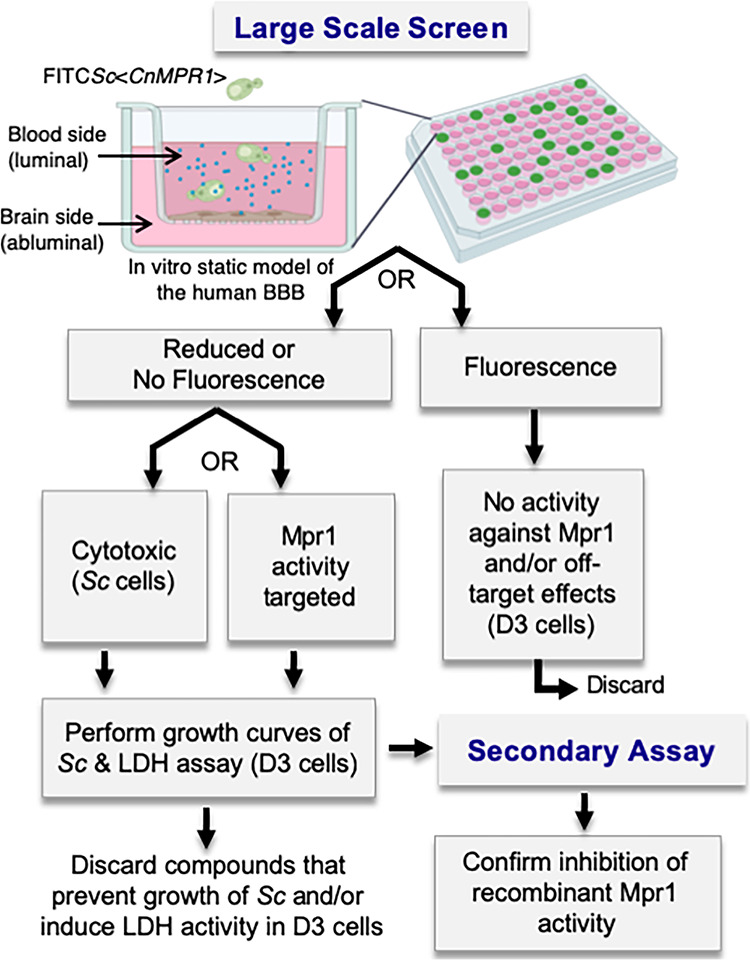
Schematic diagram of the work flow of the large-scale screen. The primary large-scale screen is performed in an *in vitro* model of the BBB in a 96-well format as a transcytosis assay. Human brain microvascular endothelial cells (hCMEC/D3 cell line, i.e., D3 cells) are grown and differentiated on a transwell insert forming a barrier that separates the luminal side from the abluminal side. An FITC-labeled *Saccharomyces cerevisea* (*Sc*) strain expressing *MPR1* cDNA (i.e., Sc<Cn*MPR1>*) from Cryptococcus neoformans is added to the top well (luminal side) in addition to a natural product library (small blue circles), and FITC-Sc<Cn*MPR1>* migration across the barrier is monitored by measuring fluorescence in the bottom well (below the transwell insert). The compounds that resulted in little to no fluorescence in the bottom well are selected and examined in the secondary assay. Here, each compound is examined in a proteolytic assay with Mpr1 recombinant protein in order to assess inhibition of Mpr1 activity.

To identify compounds that blocked the activity of Mpr1, we developed a large-scale screen (Fig. 1). The primary assay of the screen was based on previous results where we demonstrated that a wild-type strain of Saccharomyces cerevisiae (*Sc*) expressing the cDNA of *MPR1* from C. neoformans (Sc<Cn*MPR1*>) gained the ability to cross brain microvascular endothelial cells (BMECs) in an *in vitro* model of the human blood-brain barrier (BBB) (25, 32). In this simple static model, BMECs (a human CMEC/D3 [hCMEC/D3] cell line) were grown and differentiated on a transwell insert such that the upper well represented the luminal side of the BBB and the bottom well represented the abluminal (or basolateral) side of the BBB (44–46). A standard transcytosis assay involved adding fungal/yeast cells on the luminal side, collecting cells from abluminal side once they had crossed the barrier, and finally plating cells on yeast extract-peptone-dextrose (YPD) agar for CFU determination (32, 46). For the purpose of the large-scale screen, we used the Sc<Cn*MPR1*> strain in the BBB transwell assay, since it would likely enrich for inhibitors specific for Mpr1, given that Mpr1 activity alone was responsible for the ability of the Sc<Cn*MPR1*> strain to migrate across the *in vitro* BBB (25, 32).

### The large-scale screen and secondary assay.

The large-scale screen was developed as a two-tier assay (Fig. 1). In the primary screen, the *in vitro* model of the human BBB was established in a 96-well format and a fluorescein isothiocyanate (FITC)-labeled Sc<Cn*MPR1*> strain (10^4^ cells) was added to each well along with the compound library. Following a 6-h coincubation (at 37°C and 5% CO_2_), a plate reader was used to monitor fluorescence in the bottom well. Two outcomes were anticipated. (i) Detection of fluorescence in the bottom well would suggest that either the compound had zero effect on Mpr1 activity or the integrity of the barrier had been compromised. In either case, these compounds were discarded. (ii) In the second outcome, little to no detection of fluorescence in the bottom well would suggest that either the compound blocked Mpr1 activity and thus prevented Sc<Cn*MPR1*> strain from crossing the BBB, or the compound had killed the yeast strain (Fig. 1). The latter compounds were examined for their effects on yeast growth and toxicity to mammalian cells. The compounds that showed no effect on yeast growth and minimal to no cytotoxicity to hCMEC/D3 cells were further tested in the secondary assay. Here, recombinant Mpr1^HIS^ protein (47) was incubated with each lead compound, and the proteolytic activity of Mpr1^HIS^ was assessed.

### Natural product-derived compounds with drug-like qualities were screened.

**FIG 2:**
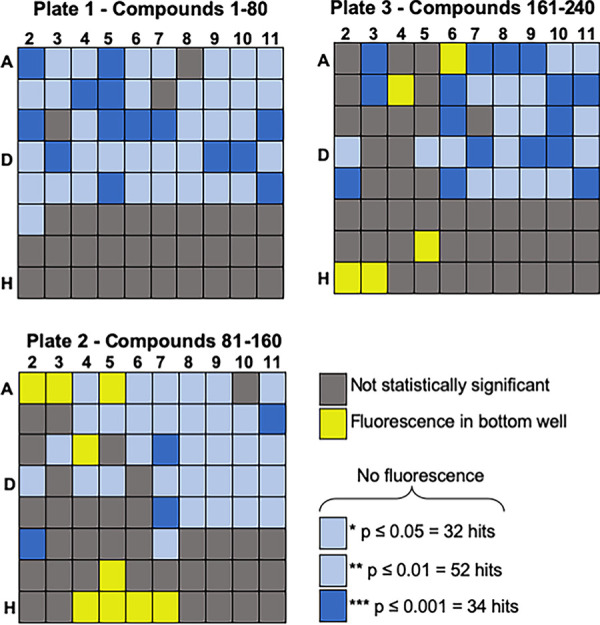
The primary screen identifies compounds that prevent the yeast strain (Sc<Cn*MPR1*>) from crossing the BBB. (A) A phytochemical-derived natural product library (*n* = 240) was screened 3 times. The fluorescence readout from the bottom well was averaged and ranked based on statistical significance. Of the 240 compounds tested, 34 compounds resulted in the greatest reduction in fluorescence (*P* ≤ 0.001, dark blue squares), 108 compounds had no effect (gray squares), and the remaining 84 compounds had variable and modest effects compared to the DMSO control.

Approximately 240 natural product-derived compounds (Prestwick, Green Pharma) were assayed three times in the large-scale screen. The FITC-labeled Sc<Cn*Mpr1*> strain was coincubated with the brain microvascular endothelial cells (hCMEC/D3 cell line) in the presence of the natural product-derived compounds (5 μM) in an *in vitro* model of the BBB for 6 h at 37°C and 5% CO_2_. After incubation, the medium in the lower transwell chamber was collected, fluorescence was measured using a microplate reader, and readings were compared to the dimethyl sulfoxide (DMSO) control. The fluorescence readout was averaged and ranked based on statistical significance (Fig. 2). Of the 240 compounds tested in the large-scale screen, 108 showed no reduction in fluorescence of the Sc<Cn*Mpr1*> strain in the bottom well (Fig. 2, gray squares). In contrast, 34 compounds showed significant reduction in the bottom-well fluorescence of the Sc<Cn*Mpr1*> strain (*P* < 0.001) (Fig. 2, dark blue squares). The six compounds that demonstrated the strongest reduction in fluorescence were selected and examined further (see Fig. S1 in the supplemental material). As expected, we found that antifungal drugs like fluconazole and ketoconazole reduced fluorescence in the bottom well, but drugs that disrupted the tight junctions of the BBB increased fluorescence compared to the that of the DMSO control (Fig. S1).

10.1128/mBio.01249-20.1 FIG S1 (A) Thirty-four out of 240 compounds showed statistically significant inhibition (*P* ≤ 0.001) of the transmigration of Sc<Cn*MPR1*>, and the top 6 inhibitors were selected for further testing (indicated by blue arrows). (B) Antifungal drugs (fluconazole, ketoconazole, and flucytosine) reduced fluorescence in the bottom well (below the transwell insert), but drugs added to the *in vitro* BBB that are known to disrupt the tight junctions of the BBB (phenylmethyl sulfonyl fluoride [PMSF] andpepstatin) produced significant fluorescence in the bottom well relative to the DMSO control. Download FIG S1, PDF file, 1.8 MB. Copyright © 2020 Aaron et al. 2020 Aaron et al. This content is distributed under the terms of the Creative Commons Attribution 4.0 International license.

**FIG 3:**
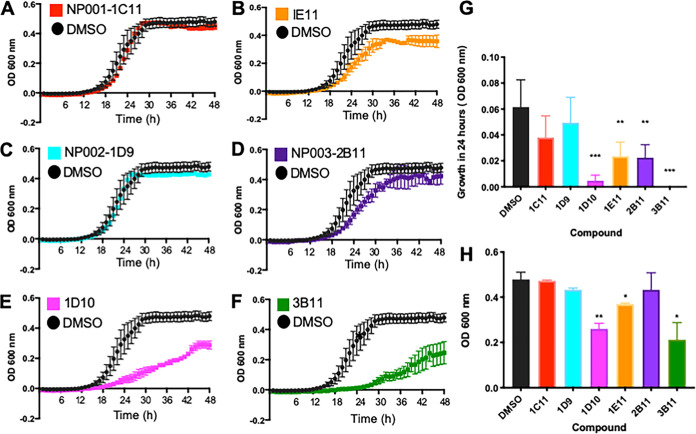
Three of the six compounds identified in the screen do not affect yeast growth. Cultures of *Sc* were treated with the top six natural products identified in the large-scale screen. Growth assays revealed that compounds NP001-1C11 and NP002-1D9 do not affect *Sc* growth (A and C), as indicated by no significant reduction of *Sc* cell density measured at 24 h (G) or 48 h (H) compared to DMSO solvent control. Compound NP003-2B11 showed no effect on *Sc* growth at 48 h but did show a slight lag at 24 h (D, G, and H). In contrast, compounds 1E11, 1D10, and 3B11 reduce growth of *Sc* compared to DMSO control (B, E, and F) indicated by the significantly lower *Sc* cell density measurements at 24 h and 48 h. Growth was assessed by measuring the OD at 600 nm over a 48-h period.

**FIG 4:**
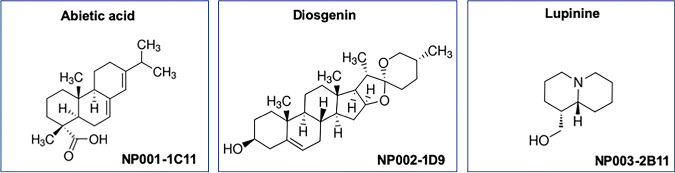
Chemical structures of the three top hits identified by the large-scale screen. The three lead compounds that satisfied all criteria of the primary and secondary assays were identified as abietic acid (NP001-1C11), diosgenin (NP002-1D9), and lupinine (NP003-2B11).

Since the aim of the large-scale screen was to identify inhibitors of Mpr1 that did not inhibit the growth/viability of *Sc*, the top six “hits” were assessed in yeast growth assays. The Sc<Cn*MPR1*> strain was coincubated with each of the six compounds, and growth of the strain was monitored over 48 h by recording the optical density at 600 nm (OD_600_) and comparing the yeast cell density at 24 h and 48 h (Fig. 3). We found that three of the six lead compounds inhibited yeast cell growth over time as indicated by the significant reduction in yeast cell density at 24 h and 48 h compared to the DMSO solvent control (Fig. 3B, E, F, G, and H). These three compounds were identified as methylxanthine (1E11), a mycotoxin (1D10), and sanguinarine (3B11). Two of the remaining three compounds had no significant effect on the growth of Sc<Cn*MPR1*> as indicated by no significant reduction in yeast cell density at 24 h and 48 h (Fig. 3A, C, G, and H). A third compound showed a slight lag in growth at 24 h, but by 48 h, no significant change in yeast cell density was observed (Fig. 3D, G, and H). The top three lead compounds that prevented fluorescence of Sc<Cn*MPR1*> strain in the bottom well and showed little to no inhibition of yeast cell growth were identified as abietic acid (NP001-1C11), diosgenin (NP002-1D9), and lupinine (NP003-2B11) (Fig. 4).

### Lead compounds block proteolytic activity of purified, recombinant Mpr1 protein.

**FIG 5:**
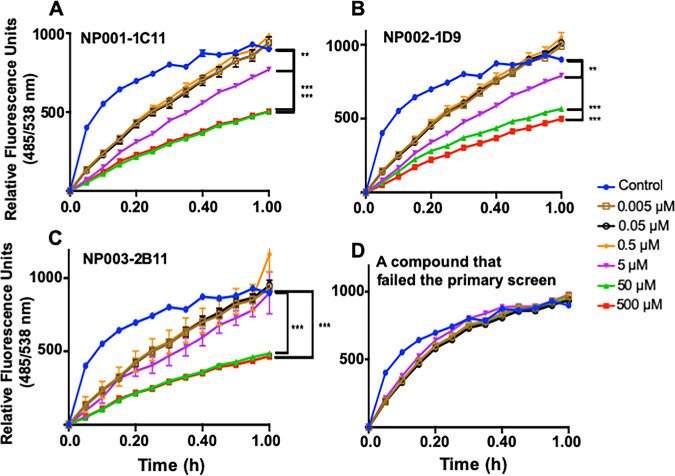
Proteolytic activity of Mpr1 protein is inhibited by the lead compounds. Increasing concentrations of the three lead compounds inhibit the activity of recombinant Mpr1 protein, and the inhibition is statistically significant compared to DMSO control (A to C). In contrast, a compound that failed the primary screen did not inhibit the proteolytic activity of Mpr1 (D). Recombinant *Cn*Mpr1-His-tagged protein was isolated via nickel-NTA magnetic beads from a culture of Pichia pastoris transformed with a plasmid expressing *Cn*MPR1-HIS cDNA as previously described (47). A FITC-labeled casein assay was used to assess the proteolytic activity of Mpr1. The Mpr1 activity in solvent (DMSO) is shown as a positive control for the proteolytic assay (blue circles). **, *P* < 0.01, ***, *P* < 0.001.

The three lead compounds satisfied the criteria for the primary large-scale screen and were therefore tested in a second assay that confirmed the inhibitory activity of the compounds. Recombinant Mpr1^HIS^ protein was isolated from the medium of a strain of Pichia pastoris expressing the *MPR1*-His-tagged cDNA from C. neoformans (47). We routinely isolate Mpr1 recombinant protein using the P. pastoris expression system—this approach produces several milligrams of active Mpr1 protein (47). In this assay, Mpr1 activity was measured using a soluble fluorescein isothiocyanate (FITC)-labeled casein enzymatic assay in the presence of the lead compounds or DMSO control (Fig. 5) (32).

**FIG 6:**
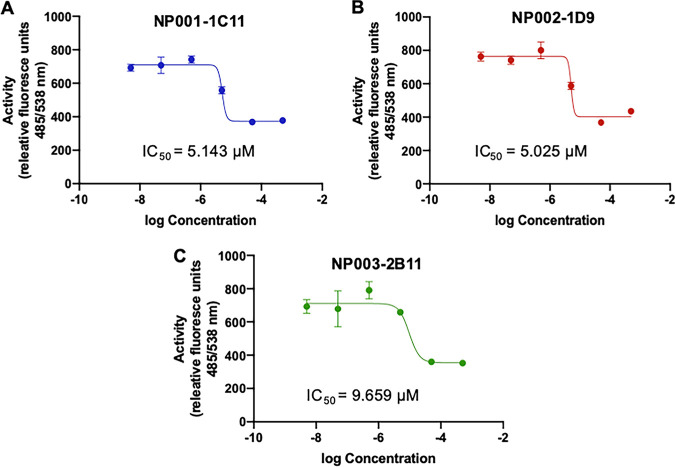
The IC_50_s for the top three compounds are less than 10 μM. The IC_50_s for NP001-1C11, NP002-1D9, and NP003-2B11 are 5.143 μM, 5.025 μM, and 9.659 μM, respectively. IC_50_ values were calculated from proteolytic activity assays similar to those shown in Fig. 5.

Increasing concentrations of the three lead compounds inhibited the activity of recombinant Mpr1 protein, and the inhibition was statistically significant compared to the control (Fig. 5A to C). In contrast, a compound that failed the primary screen did not inhibit the proteolytic activity of Mpr1 (Fig. 5D). The IC_50_ values for each lead compound were determined (Fig. 6). For all three lead compounds, the IC_50_ was less than 10 μM. In particular, the IC_50_ values for NP001-1C11, NP002-1D9, and NP003-2B11 were 5.143 μM, 5.025 μM, and 9.659 μM, respectively (Fig. 6).

### The lead compounds prevent cryptococci from crossing the BBB.

**FIG 7:**
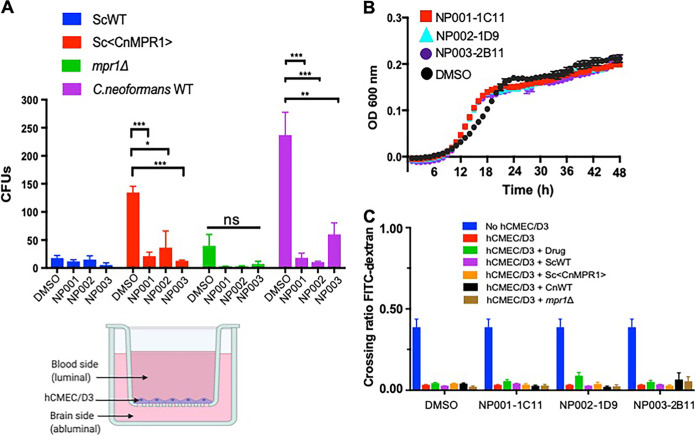
Three lead compounds prevent C. neoformans (*Cn*) from crossing the BBB. (A) Transcytosis assays 6 h posttreatment and postinoculation in a 96-well format of the *in vitro* model of the BBB (lower, right diagram). Lead compounds were tested in transcytosis assays with four fungal strains: wild-type *Sc* (ScWT), Sc<Cn*MPR1*> (expression of *CnMPR1* cDNA in *Sc*), the *Cnmpr1Δ* deletion strain, and the isogenic wild-type H99 *Cn* strain. A dose of 5 μM of each lead compound was added to the upper well. Treatment with lead compounds (NP001-1C11, NP002-1D9, and NP003-2B11) prevents crossing of *Cn* compared to DMSO control (*n* = 7, per compound). *, *P* < 0.05, **, *P* < 0.01, ***, *P* < 0.001; ns, not significant. (B) Compounds did not inhibit growth of *Cn* (*P* > 0.05). (C) Integrity of BBB was monitored by FITC-dextran (70-kDa) permeability assays in the *in vitro* BBB model. In the absence of hCMEC/D3 cells, FITC-dextran was able to freely cross the transwell (blue bar) but unable to cross when hCMEC/D3 cells were present and the BBB was intact (red bar). Treatment with compounds and addition of different strains did not promote FITC crossing, suggesting that the integrity of the BBB was not damaged by the three lead compounds.

Next, we examined whether the lead compounds could prevent *Cn* from crossing the brain endothelium using transcytosis assays in the BBB *in vitro* model (Fig. 7, bottom left). Here, a fungal/yeast strain along with the lead compounds, was added to the luminal side of human brain microvascular endothelial cells (hCMEC/D3 cell line) that were fully differentiated and recapitulated features of the human BBB (44, 46). Following a 6-h coincubation, the contents from the abluminal side of the BBB were collected and plated to quantify the migration of fungal cells across the BBB by determining the CFU. The integrity of the BBB was monitored by measuring the permeability of a 70-kDa FITC-dextran molecule, since crossing of this fluorescent probe would be indicative of BBB damage (46).

Compounds NP001-1C11, NP002-1D9, and NP003-2B11 were tested in transcytosis assays with four fungal strains: (i) the wild-type *Sc* strain, (ii) the Sc<Cn*MPR1*> strain, (iii) the *Cnmpr1Δ* deletion strain, and (iv) the wild-type H99 *Cn* strain (same parent background as the *mpr1Δ* strain). As expected, the *Sc* wild-type strain did not readily cross the BBB (*P* > 0.05, *n* = 7), similar to previous observations in our published studies (25, 32) (Fig. 7A). Importantly, the lead compounds did not appear to have off-target effects since they did not promote the migration of *Sc* across the BBB (Fig. 7A). In contrast, crossing of Sc<Cn*MPR1*> strain was significantly blocked by the lead compounds, as indicated by the reduction in CFU compared to the solvent control (Fig. 7A, *P* < 0.05, *n* = 7). This result provided further validation of the large-scale screen, since this was the same background strain used in the primary screen.

It was critical to test whether the lead compounds, NP001-1C11, NP002-1D9, and NP003-2B11 could prevent *Cn*, the causative agent of CM, from crossing the BBB. First, we examined a strain of *Cn* lacking Mpr1 (i.e., the *mpr1Δ* deletion strain) since we had previously shown that *Cn* requires Mpr1 activity to cross the BBB. As expected, *Cn* failed to cross the BBB in the absence of MPR1 (Fig. 7A) (32). Importantly, the inability of the *Cnmpr1*Δ deletion strain to cross the BBB in the presence of the lead compounds further demonstrated that the lead compounds did not produce a leaky BBB.

Most notably, we found that the addition of the three lead compounds significantly reduced the number of CFU of *Cn* (a wild-type strain of C. neoformans H99) compared to the DMSO control (Fig. 7A). This result suggested that the lead compounds blocked *Cn* from crossing the BBB; however, in order to rule out the possibility that the compounds were inhibiting *Cn* growth/viability, we examined the growth of *Cn* over a 48-h period in the presence of the three lead compounds at 5 μM. We found that the growth of *Cn* was not affected when treated with NP001-1C11, NP002-1D9, or NP003-2B11, suggesting that the lead compounds had prevented *Cn* from crossing the BBB (Fig. 7B).

In order to confirm that the integrity of the BBB was not compromised by the three lead compounds, the permeability of a FITC-dextran molecule was assessed. In the absence of cells on the transwell insert, the FITC-dextran molecule can freely move across the transwell (blue bars, Fig. 7C) but is unable to cross in the presence of the brain endothelial cells (red bars, Fig. 7C). The addition of NP001-1C11, NP002-1D9, or NP003-2B11 did not cause FITC-dextran to move across the BBB, suggesting that the BBB remained intact and that the three lead compounds did not damage the BBB (Fig. 7C).

**FIG 8:**
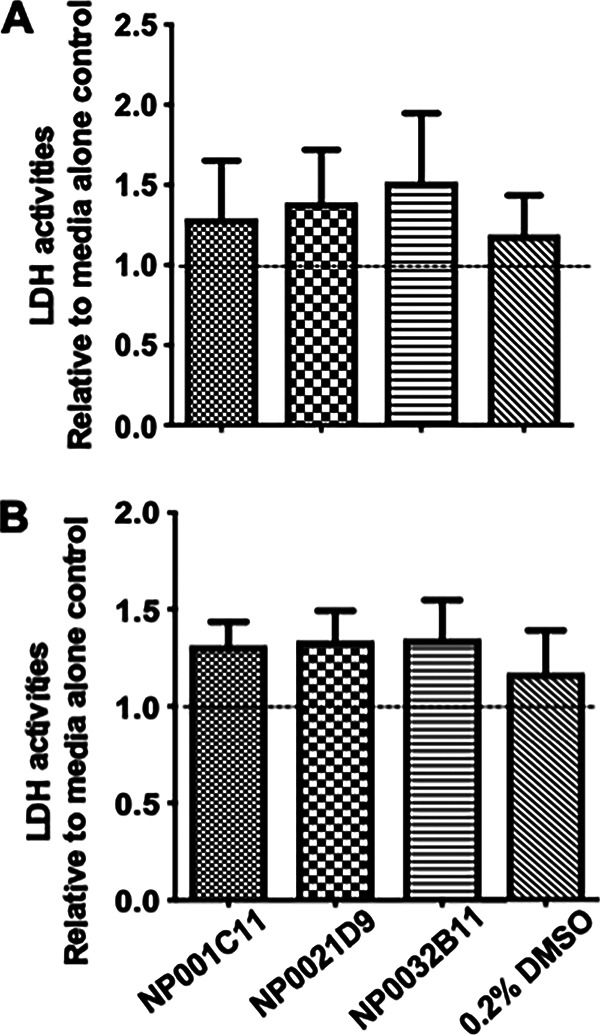
Lead compounds are not cytotoxic. Brain microvascular endothelial cells (hCMEC/D3 cell line) were treated with each lead compound. LDH, a marker of cell damage was measured at 8 h (A) and 36 h (B) posttreatment. LDH activities were not significantly different from medium/solvent control, suggesting that the lead compounds did not damage the brain endothelial cells.

Next, we assessed the cytotoxicity of NP001-1C11, NP002-1D9, and NP003-2B11 on mammalian cells. The brain microvascular endothelial cells were monitored for lactate dehydrogenase (LDH) production in the presence of the three lead compounds. LDH assays showed similar levels of LDH activity in endothelial cells treated with solvent alone or with the lead compounds for 8 h or 36 h (Fig. 8A and B).

## DISCUSSION

The goals of this study were to establish a high-throughput assay to identify small molecules that selectively inhibited Mpr1 activity and to address the notion that targeting virulence mechanisms that mediate fungal dissemination into the CNS could be a viable path forward for the development of novel antifungal agents. There is growing consensus that the current repertoire of antifungal drugs are inadequate to serve the surging challenges associated with resolving fungal infections (48). The pace of producing new antifungal drugs lags far behind with very little interest on the part of pharmaceutical companies. Part of the challenge in developing novel antifungals is the lack of viable targets in fungi, since they, like mammalian cells, are eukaryotes, and targets that are shared increase the likelihood of off-target toxicity.

The secreted metalloprotease, Mpr1, is expressed solely in fungi, and we have demonstrated its role as a virulence factor (32). We propose that Mpr1 is a high-value target of fungal virulence whose activity can be inhibited by compounds with novel structural scaffolds/features that could be further developed as antivirulence agents. The success of the large-scale screen hinged on several key components. First, a robust *in vitro* model of the human BBB where the crossing of fungal/yeast cells from the luminal to the abluminal side of the BBB could be easily monitored and quantified was required. We established the *in vitro* model of the BBB several years ago, and we developed assays that reliably examine the interactions of fungal cells with the BBB (14, 32, 46, 47). Second, it was necessary to establish the *in vitro* BBB model in a 96-well format and confirm barrier integrity so that we could establish a large-scale assay that was robust and reliable. Third, the yeast strain (Sc<Cn*Mpr1*>) was tagged with a fluorescent marker (fluorescein isothiocyanate [FITC]) which allowed detection and measurement of its transmigration across the BBB using a plate reader. The three compounds (IE11, 1D10, and 3B11) that did not satisfy the criteria of the primary screen because they prevented the growth of *Sc* further validated the screen. These compounds, methylxanthine (1E11), a mycotoxin (1D10), and sanguinarine (3B11) are known to have antifungal activity. Methylxanthine inhibits fungal chitinases, and sanguinarine has strong activity against biofilms of Candida albicans (49, 50).

The natural product compounds in the phytochemical library had several favorable features, including diversity, novelty, ease of follow-up, and agreeable pharmacological profiles. We chose to screen a natural product-derived library because our aim was to identify novel drug scaffolds that could potentially expand the repertoire of antifungal drugs, and we could build upon the biological activity of these novel structures. Also, given that fungal spores reside in soils, trees, plants, animal droppings, etc., the natural product-derived library could provide drug prototypes unique to those ecosystems.

The results provide compelling evidence that support the validity of the screen. We identified three lead compounds that inhibit Mpr1 activity, prevent *Cn* from crossing the BBB, and lack any significant cytotoxicity or off-target effects. It is unlikely that the compounds prevented *Cn* from crossing the BBB by enhancing the integrity/tightness of the BBB via endothelial cell growth, since the BBB was exposed to minimal concentrations for a short duration. There is no evidence in the literature that would suggest that the compounds promote cell differentiation or proliferation, and the compounds had no effect on the growth of *Sc* and *Cn*.

We determined IC_50_ concentrations of 5.143 μM, 5.025 μM, and 9.659 μM for the three lead compounds. This is a satisfactory starting point, and an IC_50_ of <10 μM is considered “a hit,” but ideally, a lower inhibitory concentration is preferred. We propose that the three bioactive compounds (i.e., “hits”) with distinct molecular scaffolds are viable candidates that could serve as precursors for further structural development and for *in silico* analysis to produce/identify molecular structures with more favorable kinetics (i.e., lower inhibitory concentrations and higher potency) and acceptable toxicity safety profiles.

The screen identified three compounds with very distinct chemical profiles. The compound NP002-1D9 (diosgenin) is a plant steroidal sapogenin used in traditional medicine because of several relevant biological activities, it has an agreeable safety profile, and it also serves as a precursor for the semisynthetic production of steroidal drugs (51). These features along with diverse medicinal properties, including anti-inflammatory and antiproliferative activities, have made diosgenin a promising bioactive molecule with pharmacological relevance (51). Interestingly, diosgenin has been found to inhibit the activity of matrix metalloproteinase-2 (MMP-2) and MMP-9 (52). These results are consistent with the observed inhibitory activity toward Mpr1 and suggest that diosgenin could be a feasible starting point, but further development to improve specificity would be required.

The compound NP001-1C11 (abietic acid) is an abietane diterpenoid found primarily in pine resin. Its biological activity has been largely unexplored, but derivatives of abietic acid were synthesized and examined for antimycotic and antiviral activities (53). Upon examination, some of the C18-oxygenated derivatives of abietic acid showed improved cytotoxicity (lower 50% cytotoxic concentration [CC_50_] values) and a few had antiviral activity, but none of the derivates or the parent compound displayed antimycotic activity (53). These data are in line with our observations that abietic acid did not inhibit the growth of *Sc* or *Cn*, but whether these derivatives represent more potent inhibitors of Mpr1 remains to be seen.

The compound NP003-2B11 (lupinine) is a quinolizidine alkaloid. They are secondary metabolites found primarily in the family Leguminosae, commonly referred to as the legume, pea, or bean family (54). While some quinolizidine alkaloids protect plants by acting as insecticides, little is known about the pharmacology of lupinine; however, one study found that lupinine had immunomodulatory activity on mouse splenocytes (55). Our study determined that lupinine had little to no mammalian cell toxicity, it did not damage the brain endothelial barrier, and it inhibited Mpr1 proteolytic activity with an IC_50_ of 9.659 μM. The data support further investigation of this molecular scaffold for the development of a potent inhibitor of Mpr1 and its potential use as an antivirulence agent.

Although it is interesting that the three chemically diverse compounds all inhibit Mpr1 activity with little to no off-target effects, it is perhaps not surprising, since each compound may be blocking Mpr1 activity by different mechanisms. Although we can hypothesize that the compounds are inhibiting activity by occupying the active site and excluding the substrate, by preventing the Zn^2+^ ligand from coordinating with the active site, or possibly by acting allosterically, additional studies are required in order to resolve the mechanisms of inhibition.

The human brain microvascular endothelial cells (i.e., hCMEC/D3 cell line) used in this study represent the best characterized BBB cell line, as it recapitulates features of the BBB (44). Among the many BBB features of this cell line are the expression and activity of various ABC transporters, the transferrin receptor (TfR), and P-glycoprotein transport (56). In addition, the hCMEC/D3 cell line can distinguish between molecules, as lipophilic low-molecular-weight compounds cross the hCMEC/D3 monolayer significantly better than hydrophilic molecules. Several studies have used this cell line as a model for BBB interactions with pathogens, nanocarriers, and BBB signaling (32, 45, 47, 57–59). Our studies showing that *Cn* crosses hCMEC/D3 cells by an Mpr1-dependent transcellular mechanism were supported by *in vivo* studies (32). These results further substantiate the usefulness of this cell line as a model of the BBB and also support the use of this robust BBB model here, where it was used to characterize the lead compounds as bona fide antivirulence molecules (32).

This study has shown that a drug screen can be developed to successfully identify small molecules that specifically target fungal virulence factors with little to no effect on growth/viability. We demonstrated that *Cn* was prevented from crossing the BBB by inhibiting the proteolytic activity of Mpr1 while maintaining *Cn* viability. We anticipate that the three lead compounds identified here could be further developed as antivirulence therapy that would be administered preemptively or serve as a prophylactic in patients at high risk for developing CM. The cryptococcal antigen lateral flow assay (CrAg LFA) is considered an important advancement in reducing CM-related mortality, since it has the potential to identify patients with asymptomatic infection who should receive preemptive treatments (60, 61). This test (using plasma or serum) is a dipstick immunochromatographic assay that is completed within minutes and requires no lab infrastructure (60, 61). Thus, the availability of the CrAg LFA makes antivirulence therapy a promising approach to protect high-risk populations from CM by disarming *Cn* at the early onset of infection. Alternatively, antivirulence agents that block fungal entry into the CNS could be used as adjuncts with existing antifungal agents. Our approach has focused on blocking a BBB-penetrating mechanism of pathogenicity as an antivirulence strategy against CM. Although this approach has been largely unexplored in *Cn*, a recent study demonstrated that targeting the type IV pili of the Gram-negative bacterium Neisseria meningitidis led to a reduction in meningococcal meningitis by preventing several of the hallmarks of meningococcal disease (62). Based on this study, it is conceivable that targeting mechanisms used by *Cn* to penetrate the CNS may have a profound effect on reducing the morbidity and mortality associated with CM.

In summary, the results support the use of a screen to identify small-molecule inhibitors of Mpr1 and provide a proof-of-concept that Mpr1 is a high-value target for antivirulence drug development, since Mpr1 was specifically neutralized independent of detrimental effects to *Cn* or mammalian cells and Mpr1 inhibition prevented *Cn* from crossing the BBB. Taken together, our results provide compelling evidence that a BBB-penetrating pathogenic mechanism can be blocked/inhibited independent of cell growth and also facilitate the further characterization of the lead compounds *in vivo*. Last, the study supports the notion that antivirulence approaches should be an integral part of developing novel therapies for the prevention or treatment of fungal infections.

## MATERIALS AND METHODS

### Strains and cell lines.

A wild-type strain of Saccharomyces cerevisiae (*Sc*) expressing the cDNA of *MPR1* (i.e., Sc<Cn*MPR1*>) from Cryptococcus neoformans var. *grubii* (*Cn* H99) (i.e., Sc<Cn*MPR1*>) was made as previously described (32). Briefly, the cDNA of Cn*MPR1* was subcloned adjacent to a GDP promoter in a yeast episomal 2μ expression vector and transformed into a wild-type strain of *Sc*. Fluorescein isothiocyanate (FITC) was purchased from Sigma-Aldrich, St. Louis MO.

A human brain cerebral endothelial cell line (labeled as hCMEC/D3) was obtained from B. Weksler (Cornell University) who developed and characterized this cell line as an *in vitro* model for the human blood-brain barrier (44, 45). This cell line represents a stable, fully characterized, and well-differentiated cell line that recapitulates properties of primary brain microvascular endothelial cells and has been used extensively in laboratory settings (32, 44, 46, 56). The hCMEC/D3 cell line expresses chemokine receptors, expresses unregulated adhesion molecules in response to inflammatory cytokines, and demonstrates blood-brain barrier characteristics, including tight junctional proteins and the capacity to actively exclude drugs (44, 56).

### Primary small-molecule screen.

A structurally diverse, phytochemical compound library (*n* = 240) with over 90% purity was purchased from Prestwick Chemical Library and used in the primary screen with the Sc<Cn*MPR1*> strain. The primary large-scale screen was performed in a 96-well transwell (Corning, Inc.) to replicate the *in vitro* model of the human BBB that was previously developed (32, 44, 46). Briefly, the *in vitro* BBB model consists of a transwell apparatus separating the luminal (blood) and abluminal (brain) side of the BBB. The hCMEC/D3 cells were grown in the top transwell chamber on a collagen-coated microporous membrane (8 μm). Fungal cells were grown overnight in minimal medium lacking uracil, rotating at 30°C, washed twice with 1× phosphate-buffered saline (PBS), and stained with 1 mg/ml of FITC at 37°C for 10 min, and then washed at least 5 times with 1× PBS. FITC-stained fungal cells (multiplicity of infection [MOI] = 5) were added to the top chamber of the transwell in the presence of 5 μM drug or DMSO solvent. After 6 h of incubation, 50 μl of media were collected from the top and bottom chambers and fluorescence was measured at an excitation wavelength of 485 nm and emission wavelength of 538 nm using a Spectramax M5 multilabel plate reader (Molecular Devices, Sunnyvale, CA). All compounds were tested three times, and the relative fluorescence readings (bottom well/top well) were averaged.

### *In vitro* growth kinetics of yeast and fungal cells.

Fungal cells were grown overnight in minimal medium lacking uracil and rotating at 30°C. All harvested fungal cells were washed twice with 1× PBS and added to fresh minimal medium lacking uracil (Sc<Cn*Mpr1*>) or yeast peptone dextrose (C. neoformans) containing 5 μm of drug or DMSO solvent in shaking cultures in a sealed 96-well plate at 37°C, using starting inocula at an optical density at 600 nm (OD_600_) of 0.1. OD_600_ measurements were taken every hour (with brief shaking) for 48 h using a Spectramax M5 multilabel plate reader (Molecular Devices, Sunnyvale, CA) (*n* = 3).

### Secondary assay–proteolytic activity assays.

The proteolytic activity of Mpr1 in the presence of the candidate compounds (0.005 μM, 0.05 μM, 0.5 μM, 5 μM, 50 μM, and 500 μM) or DMSO solvent was assayed using a Protease Activity assay kit (ThermoFisher Scientific, Waltham, MA) utilizing FITC-labeled casein (47). Mpr1 protein was collected from the supernatant of Pichia pastoris expressing the cDNA of Cn*MPR1-HIS* tagged and used at a concentration of 100 μg/ml as previously described (47). Briefly, a recombinant strain of P. pastoris was grown in 100 ml of BMGY medium at 30°C and 250 rpm until the culture’s OD_600_ value was between 2.0 and 6.0 (16 h). P. pastoris cells were harvested by centrifugation at 4,000 rpm at 4°C for 5 min, washed three times in BMMY medium (with 0.5% methanol and no glycerol), resuspended in BMMY at a final OD_600_ of 1.0, and incubated for 48 h at 30°C and 250 rpm. Methanol was added every 24 h at a final concentration of 0.5%. At the start of induction, Ni-NTA MagBeads (CubeBiotech, PA) were added to an P. pastoris culture. Following 48 h of induction, cultures were centrifuged at 4,000 rpm and 4°C for 10 min, and the Ni-NTA MagBeads (attached to the Mpr1^HIS^-tagged protein) were separated with a magnetic stand. The Ni-NTA MagBeads were washed (50 mM NaH_2_PO_4_, 300 mM NaCl, 20 mM imidazole) and eluted (50 mM NaH_2_PO_4_, 300 mM NaCl, 500 mM imidazole). Eluted Mpr1^HIS^ protein was concentrated and exchanged with 1× phosphate-buffered saline using 10-kDa Amicon filters (Millipore, USA), and protein concentration was measured via Bradford assay kit (ThermoFisher Scientific, Waltham, MA, USA).

Fluorescence measurements were recorded in a Spectramax M5 multilabel plate reader (Molecular Devices, Sunnyvale, CA) at an excitation wavelength of 485 nm and an emission wavelength of 538 nm at 5-min intervals for 60 min at 37°C (*n* = 3).

### Transcytosis assays in the *in vitro* model of the human BBB.

The transmigration of *Cn* in the presence of candidate compounds (5 μM) was assessed in the *in vitro* model of the human BBB with transcytosis assays as previously described (32, 46). Briefly, the *in vitro* static monolayer model of the human BBB consisted of a transwell apparatus with the lower chamber representing the abluminal (basolateral) side and the upper chamber representing the luminal (apical) side separated by a porous membrane (8 μm; Corning) (Fig. 1). The hCMEC/D3 cells used for the transcytosis assay were between passages 20 and 30. A confluent monolayer in a 25-cm^2^ culture flask was trypsinized and resuspended in 9 ml of cell culture medium. A total of 1.25 × 10^5^ hCMEC/D3 cells were seeded in rich endothelial growth medium (EBM-2; Lonza) containing growth factors and antibiotics (gentamicin and amphotericin B) on a collagen-coated transwell insert. Once added to the transwell apparatus, the hCMEC/D3 cells were cultured for approximately 2 weeks at 37°C and 5% CO_2_. During the 2-week incubation, the medium (concentration of supplements) was reduced from 1× strength on days 3 and 6 to 0.5× strength on day 9 and to 0.25× strength on day 12 and used in the assay on days 12 to 14. The use of the lower-strength medium was required to reduce the growth factors in the medium in order to promote cell differentiation and tight junction formation. The integrity of the barrier was monitored by FITC-dextran (70-kDa) permeability assays.

### Permeability assays with FITC-dextran.

Permeability across the hCMEC/D3 cells in the *in vitro* model of the BBB was assessed by the passage of FITC‐dextran (average molecular mass, 70 kDa). Following treatment of hCMEC/D3 cells, 1 mg/ml of FITC‐labeled dextran was added to each transwell. Fluorescence of FITC‐dextran was measured in samples taken from both the upper and lower wells of 96‐well black-bottom plates. Measurements were recorded at 538 nm (excitation wavelength, 485 nm) with a spectrophotometer (SpectraMax M5).

### LDH activity assays.

Cytotoxicity of the lead compounds was measured via lactate dehydrogenase (LDH) activity using the lactate dehydrogenase assay kit (Sigma-Aldrich). Cytotoxicity assays were performed with brain microvascular endothelial cells (i.e., the hCMEC/D3 cell line). Absorbance was measured at 490 nm for 30 min at 37°C, and absorbance at the highest time point was recorded before the absorbance value exceeded the maximum value obtained for the standard. LDH activity was calculated using an NADH standard set provided by the kit.

### Statistical analysis.

Data are expressed as means ± standard deviations (SD). Statistical significance was established at *P* < 0.05 (*, *P* < 0.05, **, *P* < 0.01, ***, *P* < 0.001). For the large-scale screen, *P* values of <0.001 were used to establish significance. A standard *t* test with Welch’s correction was used for comparison of two groups, and a one-way analysis of variance (ANOVA) was used when two or more groups were compared. Commercially available software (Prism GraphPad version 8.0.2) was used for statistical analyses.
