# Supplementary figures and images for: An Antivirulence Approach for Preventing Cryptococcus neoformans from Crossing the Blood-Brain Barrier via Novel Natural Product Inhibitors of a Fungal Metalloprotease
Source: mBio. 2020 Jul 21;11(4):e01249-20. doi: 10.1128/mBio.01249-20 (PMC7374060; doi:10.1128/mBio.01249-20)

**A**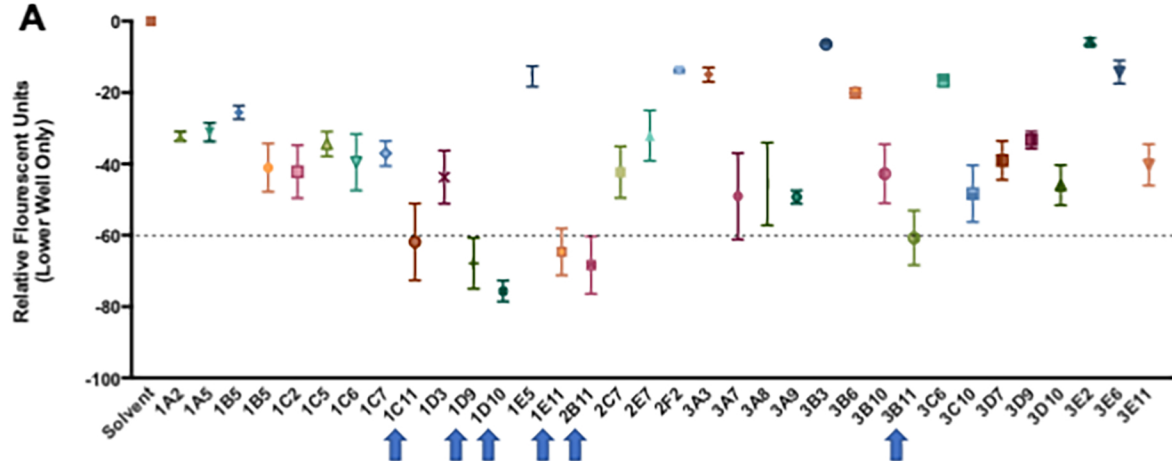**B**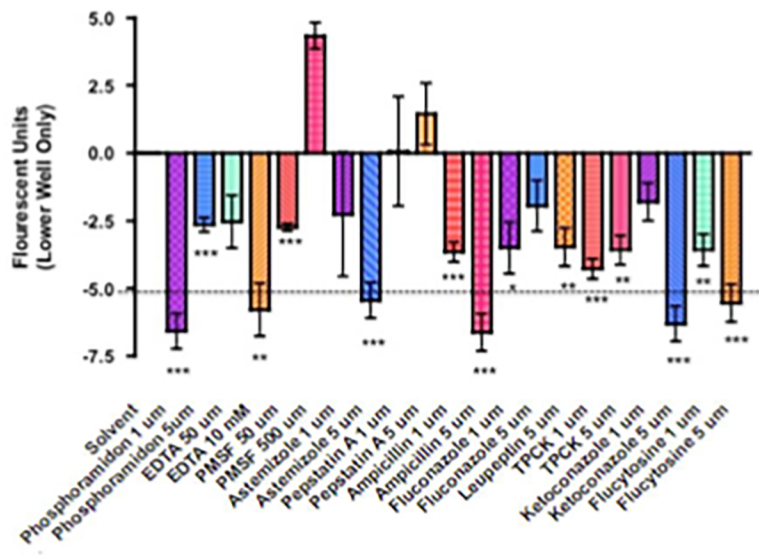

Supplement: FIG S1 [file mBio.01249-20-sf001.pdf]
